# A new species of the genus *Puto* Signoret (Hemiptera, Coccomorpha, Putoidae) from Tibet in China

**DOI:** 10.3897/zookeys.1275.179423

**Published:** 2026-03-25

**Authors:** Yu-ang Li, Xinyi Zheng, Shixiang Zong, Han Xu

**Affiliations:** 1 The Key Laboratory for Silviculture and Conservation of Ministry of Education, Beijing Forestry University, Beijing 100083, China Institute of Entomology, Guizhou University Guiyang China; 2 Institute of Entomology, Guizhou University, Guiyang 550025, China The Provincial Special Key Laboratory for Development and Utilization of Insect Resources, Guizhou University Guiyang China; 3 The Provincial Special Key Laboratory for Development and Utilization of Insect Resources, Guizhou University, Guiyang 550025, China The Key Laboratory for Silviculture and Conservation of Ministry of Education, Beijing Forestry University Beijing China

**Keywords:** Mealybugs, morphology, Putoidae, scale insects, taxonomy

## Abstract

Putoidae (Hemiptera: Coccomorpha) is a small family of scale insects in which the adult females exhibit morphological characteristics that closely resemble those of Pseudococcidae. Here we describe and illustrate the adult female and adult male of a new species, *Puto altimontanus* Li & Xu, **sp. nov**., collected from *Quercus semecarpifolia* (Fagaceae) in a high-altitude mountainous area of Xizang Autonomous Region (Tibet), China.

## Introduction

The genus *Puto* Signoret, 1876, was historically classified within the family Pseudococcidae due to the morphological resemblance of its adult females to those of mealybugs. Beardsley (1969) was the first to assign *Puto* to the family Putoidae based on adult male characteristics. Molecular evidence further supports the recognition of Putoidae as a distinct family separate from Pseudococcidae (Cook et al. 2002; Gullan and Cook 2007; Vea and Grimaldi 2016; Deng et al. 2025; Li et al. 2025). *Puto* species possess an XX-XO sex chromosome system, and the adult males possess a row of simple eyes and a single ocellus on each side of the head (Williams et al. 2011); while these features are often associated with archaeococcoids, *Puto* itself lacks other key archaeococcoid traits such as abdominal spiracles in females, and therefore does not fit neatly into either the archaeococcoid or neococcoid groups. Therefore, Deng et al. (2025) proposed that Putoidae represents a transitional group between archaeococcoids and neococcoids.

The family Putoidae currently comprises two genera and 53 species. The genus *Palaeotupo* Koteja & Azar, 2008 contains only one extinct species, *Palaeotupo danieleae* Koteja & Azar, 2008, while the genus *Puto* includes two extinct species preserved in amber, *Puto avitus* (Menge, 1856) and *Puto trivenosus* (Germar & Berendt, 1856). The remaining 50 extant species of *Puto* are distributed across the Nearctic (24 species), Palearctic (14 species), Neotropical (9 species), and Oriental regions (3 species). But only three species have been recorded in China: the Palearctic *Puto superbus* (Leonardi, 1907), and the Oriental *Puto huangshanensis* Wu, 2001 and *Puto sinensis* Zheng & Wu, 2025 (García Morales et al. 2025). A key to the adult females of Old World species is available in Zheng et al. (2025), whereas a key to the adult females of New World species can be found in Powell and Miller (2024). A key to the known adult males of *Puto* species worldwide was published by Powell and Miller (2024) and reproduced by Zheng et al. (2025) with minor revisions.

Here we describe and illustrate the adult female and adult male of a new species of *Puto* from a high-altitude mountainous area of Xizang Autonomous Region.

## Material and methods

The new species was collected in the Xizang Autonomous Region, China, on twigs and trunks of *Quercus semecarpifolia* SM. (Fagaceae). Specimens were prepared for bright-field compound microscopy using the slide-mounting method described by Kosztarab and Kozár (1988). The remaining material was preserved in 75% alcohol. The specimens are all deposited in the Insect Collection at the Department of Forestry Protection, Beijing Forestry University, Beijing, P. R. China (BFUC). The slide labels are written in Chinese.

Morphological observations were performed using a COIC ZSA0850 stereomicroscope and a Leica DM1000 compound light microscope. All measurements are presented in micrometers (μm) except for the body length and width, and lengths of antennae and fore wings in the adult male, which are given in millimeters (mm). The morphological terms for adult females used in our descriptions mainly follow Williams (2004); for the adult male, the terminology is based on Hodgson and Foldi (2006), with wing venation terms following Wu and Xu (2022). Dorsal oral-rim tubular ducts of *Puto* have been referred to by various names in past studies, such as “oral-rim type tubular ducts”, “large tubular ducts”, “enlarged tubular ducts” and “wide ducts” (McKenzie 1967; Williams and Granara de Willink 1992; Williams et al. 2011; Danzig and Gavrilov-Zimin 2014; Powell and Miller 2024). In this paper, we use the term “oral-rim type tubular ducts” to describe the ducts associated with the cerarii, those on the dorsum, and those on the ventral abdomen (only in the description). However, the tubular ducts located anterior to the mouthparts on the venter are distinctly different from these and are described herein as oral-collar tubular ducts; the latter ducts were discussed by Williams et al. (2011). Following the description provided by Tang (1992) for cerarii of Pseudococcidae, the “basic pairs” are defined as the 18 pairs typically present in a standard complement: 4 pairs on the head, 6 pairs on the thorax, and 8 pairs on the abdomen. Additional cerarii refer to structures located between the 18 basic pairs or present on the submargin or dorsum. They are composed of several trilocular pores and a few lanceolate setae.

## Results

### Taxonomy

#### Genus Puto

Taxon classificationAnimaliaHemipteraPseudococcidae

Signoret, 1876

03331B7A-77CD-5097-853B-39625C0EC88C

Putonia Signoret, 1875: 341, type species Putonia antennata Signoret, 1875; junior homonym (previously used in Heteroptera by Stål 1872).
Puto Signoret, 1876: 394 (replacement name for Putonia Signoret, 1875).
Macrocerococcus Leonardi, 1907: 151, junior subjective synonym discovered by Danzig 1980: 110.

#### Puto altimontanus

Taxon classificationAnimaliaHemipteraPseudococcidae

Li & Xu sp. nov.

5588748A-BDD1-5BCA-8C6F-8C63B2049C7E

https://zoobank.org/A87F8D31-8CC3-4B42-9D69-3BCA50267007

##### Material examined.

***Holotype***: China • ♀; Xizang Autonomous Region, Linzhi City, Bayi District, Lulang International Tourism Town; 29°44'03"N, 94°43'21"E, altitude 3300 m; 11 August 2025; Yu-ang Li leg; on *Quercus semecarpifolia* (Fagaceae); BFUC. Holotype mounted together with 1 paratype ♀ on 1 slide; holotype specimen is indicated with a circle in permanent marker on the coverslip. ***Paratypes***: China • 7 ♀♀ • 4 ♂♂; same data as holotype: 1 ♀ mounted together with holotype on 1 slide, 4 ♀♀ mounted singly on 4 slides, and 2 ♀♀ mounted together on 1 slide; 2 ♂♂ mounted singly on 2 slides, and 2 ♂♂ mounted together on each of 2 slides; 4 male forewings (from 2 of the above males) mounted together on 1 slide.

##### Etymology.

The specific name is a masculine adjective formed by the combination of the Latin prefix ‘*alti-*’, meaning high as in *altus*, the root ‘-*mont*-’, meaning mountain as in *mons*, and the suffix ‘-*anus*’, meaning pertaining to. The name alludes to the high-altitude mountainous habitat of this species.

##### Description.

**Figure 1.**
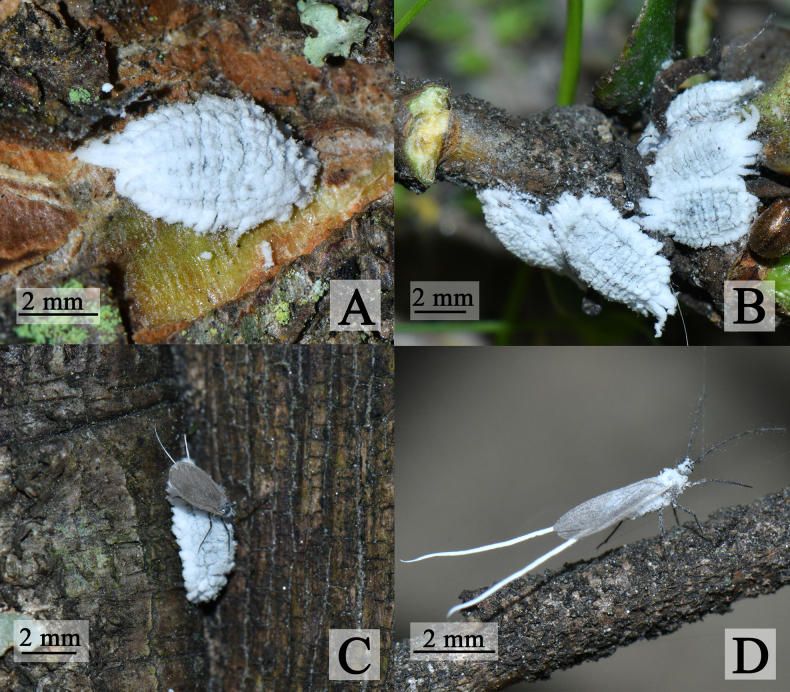
Habitus photographs of *Puto altimontanus* Li & Xu, sp. nov. **A**. Adult female; **B**. Habitat of the type samples; **C**. Adult male and female; **D**. Adult male.

**Adult female. *Appearance in life*** (Fig. 1A–C). Body oval, dorsum densely coated in white wax, marginally extended into wax plates (approximately 15 or 16 pairs), venter thinly covered with mealy wax.

**Figure 2.**
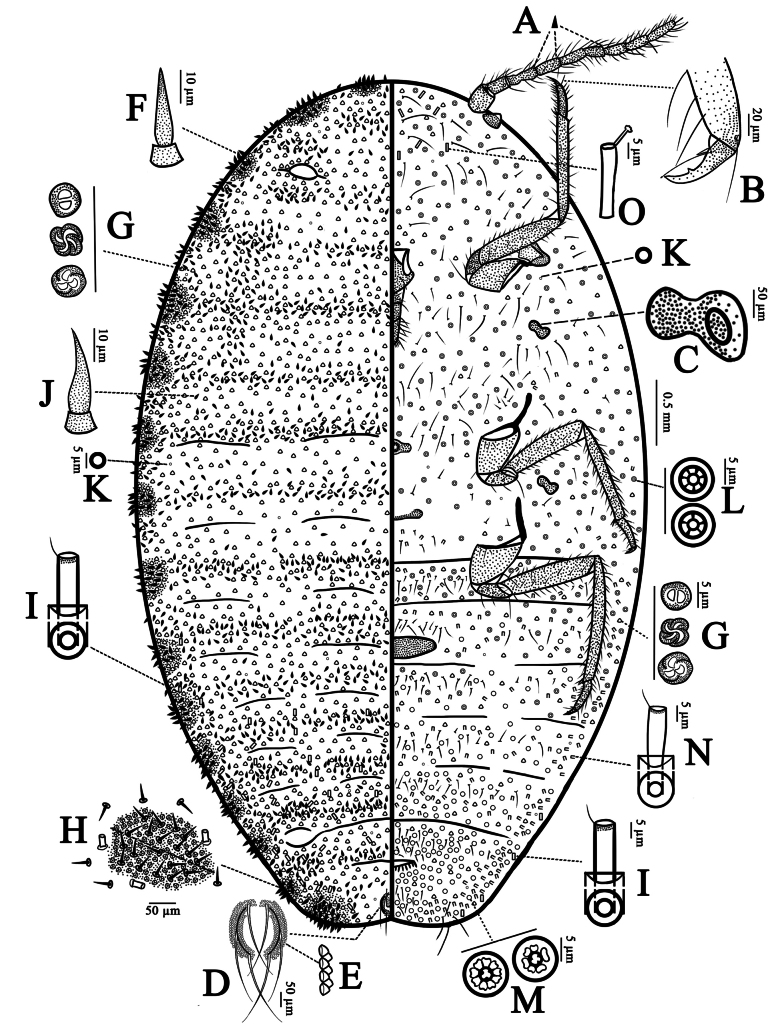
Adult female of *Puto altimontanus* Li & Xu, sp. nov. **A**. Intersegmental sensillum; **B**. Claw; **C**. Thoracic spiracle; **D**. Anal ring; **E**. Conical cells of anal ring (lateral view); **F**. Lanceolate cerarian seta; **G**. Trilocular pore (also sometimes with two or four loculi); **H**. Anal-lobe cerarius; **I**. Large oral-rim type tubular duct; **J**. Lanceolate seta on dorsum; **K**. Discoidal pore; **L**. Small multilocular disc-pores; **M**. Large multilocular disc-pores; **N**. Small oral-rim type tubular duct; **O**. Slender oral-collar tubular duct.

***Slide-mounted material*** (*N* = 7) (Fig. 2). Body 6.2–6.8 mm long and 3.3–3.5 mm wide. Antennae 9-segmented, each 1539–1662 μm long; segment III longest, segment lengths (in µm): I, 163–175; II, 150–193; III, 235–238; IV, 160–183 V, 175–200; VI, 148–150; VII, 150–155; VIII, 163–168; and IX, 195–200. Each segment nearly cylindrical; antenna with many hair-like setae, each 88–125 μm long. Scape widest, 150–175 μm wide, with 5 hair-like setae; pedicel (segment II) 75–90 μm wide, with 1 circular sensory pore and 2 coeloconic sensilla; remaining segments each 58–68 μm wide. Segments III, IV and VI each with a pair of intersegmental sensilla (Fig. 2A) near distal end; segments VII and VIII each with a sensory seta near apex, apical segment (IX) with 2 sensory setae, each 50–65 μm long. Eyes protruding, 105–123 μm high, and are situated posterior to the scape. Each eye has a sclerotized base and an apex that is 68–73 μm in diameter with a membranous margin. Clypeolabral shield 420–480 μm long, posterior part bearing a pair of setae, each seta about 125–135 μm long. Labium 3-segmented, 385–405 μm long; segment I membranous, bearing 1 hair-like seta (103–115 μm long) and 2 spine-like setae (38–50 μm long) on each side; segments II and III sclerotized, segment II with 1 hair-like seta (88–98 μm long) on each side; segment III with many hair-like setae, each 63–138 μm long, and shorter setae near apex, each 13–35 μm long. Legs well developed, forelegs and middle legs subequal in length, hind legs longest; lengths (in μm): foreleg: entire length 2203–2313; coxa 260–280; trochanter + femur 830–860; tibia 810–850; tarsus 225–240, and claw 78–83; middle leg: entire length 2235–2340; coxa 300–310; trochanter + femur 840–890; tibia 790–810; tarsus 225–245, and claw 80–85; hind leg: entire length 2635–2795; coxa 330–350; trochanter + femur 950–1010; tibia 1000–1070; tarsus 275–280, and claw 80–90. Hind leg tibia + tarsus 1.3 times as long as trochanter + femur; tibia 3.6–3.8 times as long as tarsus. Legs each with many hair-like setae, each 53–115 μm long; translucent pores absent. Trochanters bearing 3 or 4 sensory pores (campaniform sensilla) on each surface (with 3 being the most common number) plus a long hair-like seta, 210–275 μm long. Tarsal digitules each 83–95 μm long, with pointed apex. Claws (Fig. 2B) each with a plantar denticle and a pair of basal spurs; claw digitules each 62–66 μm long, with capitate apex, longer than claw. Opening of thoracic spiracles (Fig. 2C) each 113–143 μm in maximum width. Circulus complete, elongate oval, 590–600 μm wide, more than 3 times as wide as long, located on abdominal segment III. Anterior and posterior ostioles present, each with inner edges of lips sclerotized; each lip with numerous trilocular pores, without oral-rim tubular ducts, oral-collar tubular ducts and lanceolate setae; outer limits of lips not well defined. Anal ring (Fig. 2D) 150–160 μm in diameter, with 2–5 rows of conical cells (Fig. 2E) and bearing 6 setae (some setae bifurcated in paratypes), each 350–360 μm long. Cerarii numbering 16 basic pairs; each cerarius situated on a sclerotized area, with many lanceolate setae (Fig. 2F), each seta 29–32 μm long and about 5 μm wide, and many trilocular pores (also with 2 or 4 loculi in some instances, all of comparable size to typical trilocular pores) (Fig. 2G), each pore 6–7 μm in diameter. Anal-lobe cerarii (Fig. 2H) each containing 21–25 lanceolate setae and 52–65 trilocular pores; oral-rim type tubular ducts (Fig. 2I) present near anal-lobe cerarii.

***Dorsum***. Dorsal surface with numerous lanceolate setae (Fig. 2J) present, each 23–31 μm long and about 4–5 μm wide; forming groups on head and in submarginal area of thorax, and in a transverse band on all segments. Multilocular disc-pores absent. Trilocular pores (Fig. 2G) each 5–8 μm in diameter (occasionally with 2 or 4 loculi), evenly scattered over entire dorsum. Oral-rim type tubular ducts (Fig. 2I) each 17–23 μm long and 5–6 μm wide, present around each of last 7 cerarii, with about 0–2 ducts present on head (not associated with cerarii); also sparsely distributed across abdominal segments IV–VII. Discoidal pores (Fig. 2K) each about 5 μm in diameter, very sparsely scattered on the entire dorsum.

***Venter***. Ventral surface with normal flagellate setae present, each 50–173 μm long, scattered on head and thorax; forming a transverse band each across abdominal segment, and dense around vulva. Multilocular disc-pores scattered across all segments and of 2 sizes: (i) small disc-pores (Fig. 2L), each 8–9 μm in diameter, with 5 or 7 (mostly 7) quadrilateral-shaped loculi, present from head to abdominal segment IV, reaching almost to margins; and (ii) large disc-pores (Fig. 2M), each 10–11 μm in diameter, with 6 or 9 (mostly 9) kidney-shaped loculi arranged around a rounded-triangular central area, present from abdominal segment III to posterior of abdomen, reaching almost to margins, numerous around vulva. Trilocular pores, each 7–8 μm in diameter, evenly distributed over the entire ventral. Oral-rim type tubular ducts of 2 sizes: (i) large oral-rim type tubular ducts, same size as ducts on dorsum, a few on margins of abdomen segments VI–VIII; and (ii) small oral-rim type tubular ducts (Fig. 2N), each 18–20 μm long and 4–5 μm wide; forming transverse bands on abdominal segments II–VI, and forming groups on the margins of abdominal segments VII and VIII scattered on metathorax and submargin of mesothorax. Oral-collar tubular ducts (Fig. 2O) of 1 size: slender ducts, each 23–25 μm long and 3–4 μm wide, with 10–15 ducts present on head, not reaching level of clypeolabral shield. Each anal lobe with 1 anal-lobe seta, each 180–220 μm long. Discoidal pores extremely sparse and scattered, as on the dorsum, each about 5 μm in diameter.

***Diagnostic characteristics***. (i) circulus on abdominal segment III complete, more than three times as wide as long; (ii) small multilocular disc-pores abundant from head to abdominal segment IV; (iii) large multilocular disc-pores present from abdominal segment III to posterior end of abdomen; (iv) ostioles without setae on lips; (v) on dorsum, oral-rim type tubular ducts present around last seven cerarii, and across abdominal segments IV–VII; (vi) on venter, a few oral-collar tubular ducts present on anterior of head, and small oral-rim type tubular ducts present on abdomen and parts of thoracic segments II and III; and (vii) cerarii numbering 16 pairs.

***Remarks***. The adult female of *P. altimontanus* is closest to that of *P. thailandicus* Williams, 2004, based on a comparison using the key to *Puto* from the Old World by Zheng et al. (2025), in having the following character states: (i) oral-collar tubular ducts absent from cerarii; (ii) dorsum without additional dorsal cerarii; and (iii) venter without lanceolate setae. But *Puto altimontanus* differs from *P. thailandicus* by having (contrasting character states in *P. thailandicus* given in parentheses): (i) cerarii numbering 16 basic pairs (cerarii probably numbering 17 basic pairs); and (ii) multilocular disc-pores of 2 sizes, each pore with 5, 6, 7 or 9 subcentral loculi only (with multilocular disc-pores of only 1 size, each pore with 8 subcentral loculi) (data of *P. thailandicus* are derived from Williams (2004)).

In China, the adult female of *P. altimontanus* is also similar to the *P. huangshanensis* Wu, 2001, in having the following character states: (i) dorsum without additional dorsal cerarii; (ii) oral-rim tubular ducts absent from cerarii; (iii) circulus on abdominal segment III more than three times as wide as long; and (iv) anal ring bearing six acute setae. *Puto altimontanus* differs from *P. huangshanensis* by having (contrasting character states in *P. huangshanensis* given in parentheses): (i) venter without lanceolate setae (lanceolate setae present on venter); and (ii) ostioles without setae on lips (ostioles with setae on lips) (data of *P. huangshanensis* are derived from Zheng et al. (2025)).

Among the Chinese species, the adult female of *P. altimontanus* differs from *P. sinensis* by having (contrasting character states in *P. sinensis* given in parentheses): (i) large disc-pores present from abdominal segment III to posterior of abdomen (large disc-pores present only on posteriormost 3 abdominal segments); (ii) ostioles without lanceolate setae on lips (ostioles with setae on lips); and (iii) cerarii numbering 16 pairs (cerarii numbering 17 pairs) (data of *P. sinensis* are derived from Zheng et al. (2025)).

**Adult male. *Appearance in life*** (Fig. 1C, D). Body midge-like; forewings gray; body coated with white wax; posterior abdomen with 2 long wax filaments, approximately 1.3 times body length.

**Figure 3.**
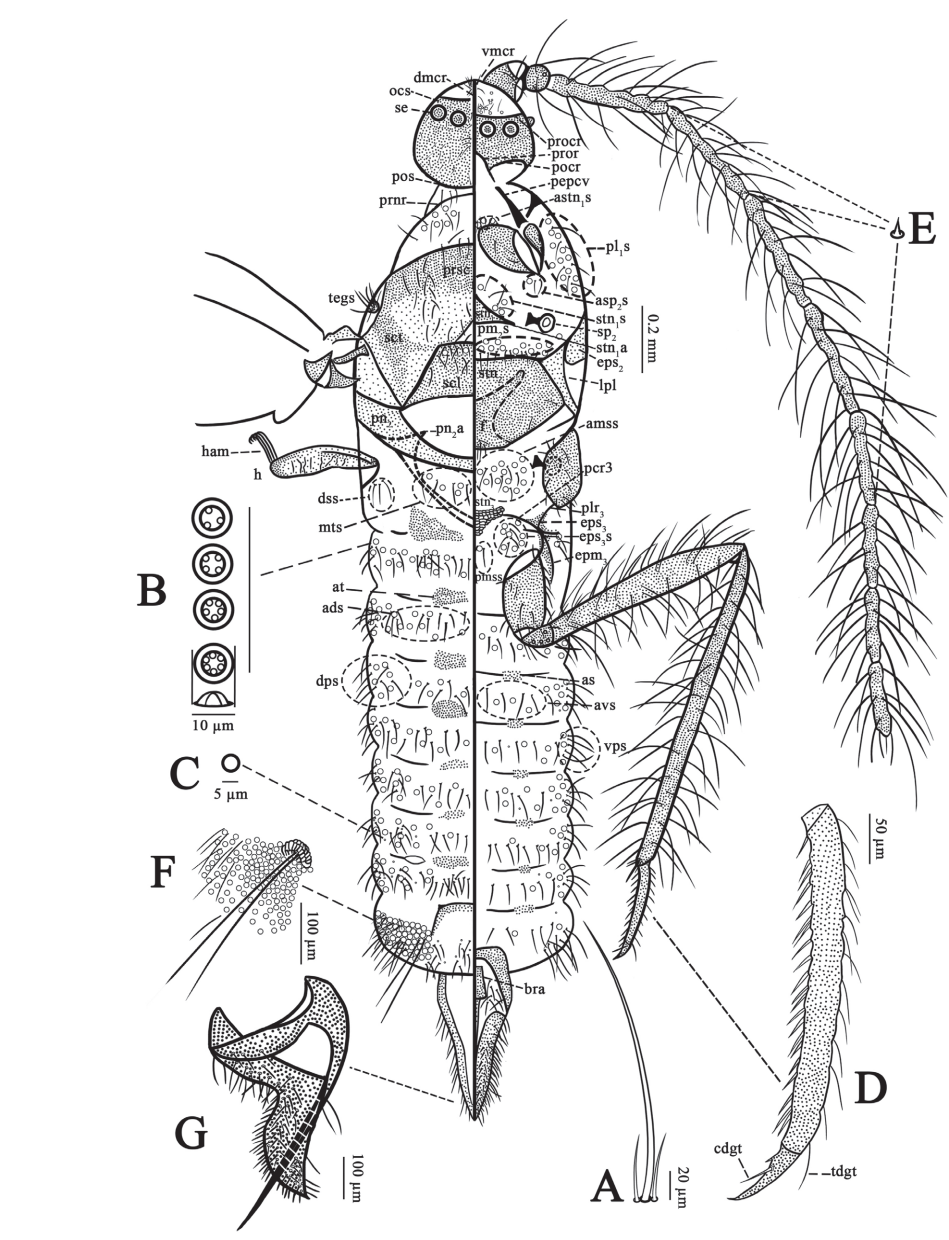
Adult male of *Puto altimontanus* Li & Xu, sp. nov. **A**. Long hair-like seta with satellite setae; **B**. Loculate pores; **C**. Small simple pores; **D**. Tarsus and claw; **E**. Antennal intersegmental sensillum; **F**. Glandular pouch; **G**. Penial sheath in lateral view.

***Slide-mounted material*** (*N* = 4) (Fig. 3). Body large, total body length about 2.9–3.1 mm. Head with large ocular sclerites (ocs), each with 5 simple eyes (se). Antennae long, more than half of total body length, each with long hair-like setae (hs) and satellite setae (Fig. 3A) present. Body with many long hs. Loculate pores (lp) (Fig. 3B) present on venter of head; on dorsum and venter of thorax and abdomen, each 8–10 μm wide, with mostly 4 or 5 loculi (rarely 3 or 6 loculi). Small, simple pores (smp) (Fig. 3C) present, each about 5 μm in diameter, scattered around lp. Legs well developed; long setae on trochanter, femur and tibia with satellite setae, without bifurcated setae; tarsi (Fig. 3D) 2 segmented; claws each with a pair of basal spurs, plantar denticle weak, plus a weak denticle near apex; claw digitules each with a pointed apex. Wings without alar setae or sensoria. A pair of ostioles present between abdominal segments VI and VII; and a pair of glandular pouches present on abdominal segment VIII. Aedeagus slender.

***Head*** 340–370 µm long, 380–410 µm wide; sclerotized ocs extends as a wide band around head, each side with a row of 5 se. Dorsum: dorsal mid-cranial ridge (dmcr) extending full length of head, fusing with transverse postoccipital suture (pos) posteriorly; 26–36 dorsal head setae (dhs) present on each side of dmcr, each 28–33 µm long; lp absent. Laterally: Ocular sclerite (ocs) heavily sclerotized, not reticulated, each ocs with a transverse band of 5 se, those near mid-cranial ridge larger, each 40–45 µm in diameter; lateral se smaller, 33–35 µm in diameter; se prominent. Ocellus (o) not visible. Ventrally: Ventral arm of mid-cranial ridge (vmcr) extending to fuse with preoral ridges (pror) posteriorly. Preocular ridge (procr) fusing with vmcr medially. Area anterior to procr membranous, with 14–22 ventral head setae (vhs), each 35–88 µm long, 7–10 lp and about 1–3 smp. Postocular ridge (pocr) developed, extending from pror posteriorly along posterior margin of ocs ventrally. Preoral ridge (pror) sclerotized, extending from posterior end of vmcr outwards and posteriorly, articulating with cervical sclerites (pepcv). Area posterior to pror membranous, with a central mouth (m).

***Antennae*** 10-segmented, each 2.5–2.8 mm long, ratio of total body length to antennal length 1: 0.8–0.9. segment length (in µm): I, 80–120; II, 60–80; III, 330–380; IV, 330–350; V, 320–460; VI, 340–370; VII, 280–340; VIII, 250–300; IX, 255–260; and X, 210–240. Scape (scp) with a strongly sclerotized basal ring for articulation with head, 110–113 µm wide, with 7–10 hs. Pedicel (pdc) 75–80 µm wide, completely sclerotized, with 5 or 6 long setae (each bearing 0–2 satellite setae), 3 or 4 short hs plus a campaniform sensillum (camp). Each segment of flagellum parallel-sided, 43–50 μm wide, with many long hs, most bearing 1 or 2 satellite setae; each long hair-like seta 188–200 μm long. Segments VIII and IX each with 1 sensory seta near apex; apical segment with 2 sensory setae subapically; each sensory seta 80–100 μm long. Antennal intersegmental sensilla present between segments III–IV, IV–V and VII–VIII, with 2 sensilla at each location (Fig. 3E).

***Thorax. Prothorax: Dorsum***: pronotal ridges (prnr) well developed and fused medially, extending ventrally and nearly touching proepisternum + cervical sclerite (pepcv); with 1–3 hs anteriorly on each side between prnr and pos. Pronotal sclerite (prn) absent. With 10–14 long setae immediately behind prnr, and 4–6 lp on each side. Post-tergites (pt) absent. Laterally: proepisternum + cervical sclerite (pepcv) anterior part articulating with pror, posterior end heavily sclerotized and broad, articulating with prnr and coxa. With a large group of 14–19 long propleural setae (pl_1_s) plus 18–22 lp. Venter: sternum (stn_1_) with strong transverse and median ridge; sternite sclerotized; prosternal apophyses (stn_1_a) developed; each side of sternum with 4 or 5 prosternal setae (stn_1_s), 3 or 4 lp, and 0 or 1 smp. Near procoxa with a group of about 4 anteprosternal setae (astn_1_s), 5 or 6 lp; and a group of 0–3 antemesospiracular setae (asp_2_s), 0–2 lp anterior to each anterior spiracle.

***Mesothorax: dorsum***: prescutum (prsc) sclerotized but poorly separated from scutum (sct), prescutal ridge and suture absent; without prescutal setae. Scutum (sct) 570–620 µm wide, bearing 2 longitudinal bands of 14–22 long setae on its lateral areas and 2 or 3 long setae on each lateral margin. Scutellum (scl) 210–250 µm wide, 130–140 µm long, with well-developed marginal ridges, and 11–13 scutellar setae (scls) and 4 or 5 lp. A small triangular membranous area present immediately anterior to scl, about 40–45 µm long and 140–150 µm wide, without setae. Laterally: prealare (pra) sclerotized. Mesepisternum (eps_2_) not reticulated; subepisternal ridge (ser) developed. Tegula (teg) with 9–11 tegular setae (tegs). Mesothoracic spiracles (sp_2_) each with outer part of peritreme 53–55 µm wide. Venter: basisternum (stn_2_) 450–500 µm wide, 275–290 µm long; without median ridge (mdr); bounded anteriorly by a developed marginal ridge (mr), basisternum bounded posteriorly by well-developed mesoprecoxal ridges (pcr_2_); additional short ridge extending ventrally from pcr2 near articulation of mesocoxa; stn2 with 9–12 basisternal setae (stn_2_s). Lateropleurite (lpl) narrow, area near stn2 weakly sclerotized. Furca (f) with long arms, each arm about 150–170 µm long. Mesopostnotum (pn_2_) and postnotal apophyses (pn_2_a) well developed. Postmesospiracular setae (pm_2_s) with 0 or 1 long seta medially; posterior to each spiracle with a group of 7–10 long setae, 10–15 lp, and about 2 smp.

***Metathorax: dorsum***: metapostnotum (pn_3_) either absent or fused to abdominal tergites of abdominal segment I, with 9–12 metatergal setae (mts), 5–7 lp and 0 or 1 smp. Dorsospiracular setae (dss): 3–5 long setae present. Laterally: metapleural ridge (plr_3_) well developed, with small suspensorial sclerites (ss); episternum (eps_3_) weakly sclerotized, with a group of 2–7 postmetaspiracular setae (eps_3_s), 5–8 lp and 0 or 1 smp. Precoxal ridge (pcr_3_) developed, extending ventrally, 150–175 µm long; plr_3_ with an additional short sclerotization extending ventrally close to coxal articulation. Metepimeron (epm_3_) extending posteriorly. Venter: metathoracic spiracles (sp_3_) each with outer part of peritreme 75–80 µm long, with 8–11 antemetaspiracular setae (am_3_), 9–11 lp and 1 or 2 smp. Metasternum (stn_3_) weakly sclerotized and reticulated, with lateral metasternal apophyses (stn_3_a); with about 19 anterior metasternal setae (amss), 6–8 lp and 1 or 2 smp; and with about 2 posterior metasternal setae (pmss), 0–3 lp; stn3a posteriorly present near a group of 8–10 long setae and 2–4 lp. Postmesoprecoxal ridge setae (ppcr_2_s) absent.

**Figure 4.**
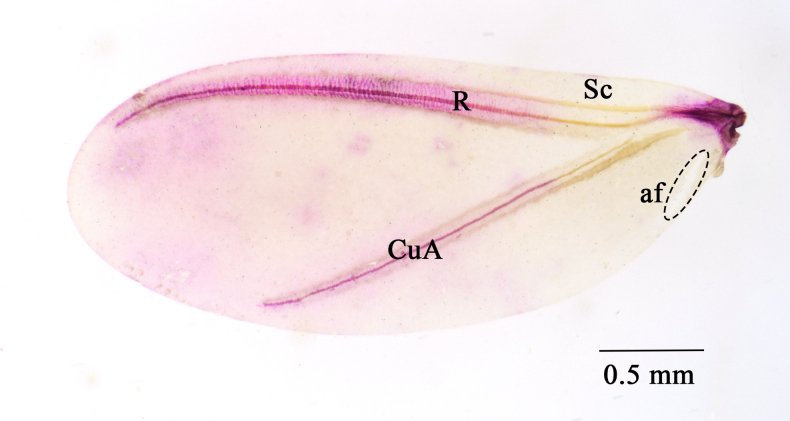
The wing of *Puto altimontanus* Li & Xu, sp. nov. adult male paratype. **Sc**, subcostal; **R**, radius; **CuA**, cubitus anterior; **af**, anal fold.

***Wings*** (Fig. 4). Forewings oblong and well developed, hyaline, 2.7–3.2 mm long, 1.2–1.4 mm wide; ratio of length to width 1: 0.4, and ratio of total body length to wing length 1:0.9–1.0; covered in microtrichia; without alar setae (als) or sensoria (sens); alar lobe (al) present. Subcostal (Sc) arising from alar sclerites; radius (R) joining subcosta; cubitus anterior (CuA) present, not joined with other veins, with a light pigmented band above and dark pigmented band below; anal fold (af) transparent. Hamulohalteres (h) each 225–248 µm long and 30–55 µm wide; with anterior margin sclerotized and 4 hamuli (ham) at tip, each with apex curved, 113–128 µm long.

***Legs*** well developed; lengths (in µm): foreleg: entire length 2680–2980; coxa 200–210; trochanter + femur 680–760; tibia 840–920; tarsus 260–340; claw 70–75; middle leg: entire length 2610–2890; coxa 230–250; trochanter + femur 640–730; tibia 790–900; tarsus 300–330; claw 65–68; hind leg longest: entire length 2705–3250; coxa 200–250; trochanter + femur 650–800; tibia 920–1050; tarsus 310–350; and claw 63–80. Ratio of length of metatibia to length of metatarsus 1: 0.4. Each leg with many long hs, each seta 138–153 µm long; trochanter, femur and tibia with satellite setae; each coxa with some short hs, each 30–68 µm long, without satellite setae. Trochanters with 4 sensory pores on each surface. Tibia with some spine-like setae along its inner margin from mid-length to apex, each seta 35–40 µm long. Tarsi each 2 segmented, segment I triangular, without setae; segment II with a tarsal campaniform sensillum proximally, outer side with short hs, each 25–50 µm long, inner side with spine-like setae, each 28–40 µm long; tarsal digitules (tdgt) with pointed apices, each 63–65 µm long. Claws each with a pair of basal spurs, each 9–10 µm long, plantar denticle weak or absent, a weak denticle near apex; claw digitules (cdgt) conical, each 28–33 µm long, shorter than claw.

***Abdomen***. Segments I–VII: tergites (at) represented by pairs of sclerotizations on anterior margins of all segments; sternites (as) weakly sclerotized. Caudal extensions (ce) absent. A pair of ostioles present laterally between segments VI and VII. Segments I–VII with 10–24 dorsal abdominal setae (ads), 8–13 lp and 0–3 smp. Pleural setae: dorsopleural (dps) and ventral pleural setae (vps) present on each side, with 9–13 long setae, 10–15 lp and 0–2 smp. Ventral abdominal setae (avs): 12–16 long setae, 4–6 lp and 0–2 smp. Segment VIII with a strongly sclerotized and slightly convex area across segment, a group of ads posterior to at and a small, weakly sclerotized as with a group of avs posterior to it. Glandular pouches (gp) (Fig. 3F) very large, each with 145–166 lp spreading out around glandular pouch, pores mostly with 4 or 5 loculi, rarely 3 or 6 loculi; each pouch with 2 rather thick glandular pouch setae (gls), each 240–328 µm long, plus 19–21 shorter setae, each 48–75 µm long; each pair of gls with a large group of duct-like pores around their base.

***Genital segment***: anus (an) not visible. Penial sheath (ps) (Fig. 3G) fairly broad basally, tapering to a point; 490–500 µm long, maximum width about 215 µm; mostly membranous but with a sclerotized framework. Membranous surfaces of dorsum and venter with many hs; sclerotized framework with some hs anteriorly, becoming spine-like and shorter posteriorly; with sensoria (psp) near tip. Basal rod (bra) sclerotized, 150–215 µm long. Aedeagus (aed) sclerotized, 650–700 µm long, apex curved, broadest at base, tapering posteriorly; attached to bra basally.

***Diagnostic characteristics***: (i) forewings developed; (ii) tarsal digitules each with pointed apex (iii) claws each with a pair of basal spurs, plantar denticle weak or absent, with a weak denticle near apex; (iv) with five eyes on each side of head; (v) tegula with 9–11 tegular setae; and (vi) apex of aedeagus not bilobate.

***Remarks***. The adult male of *P. altimontanus* differs from that of many *Puto* species by having five eyes on each side of the head. Most previously described adult males of *Puto* possess six or seven pairs of simple eyes, except the adult male of *P. superbus* has four pairs of simple eyes (Williams et al. 2011). The descriptions of males for *P. antioquensis* (Murillo, 1931) and *P. simmondsiae* McKenzie, 1961 could not be found. Williams et al. (2011) recorded five pairs of simple eyes for the male of *P. pacificus*. Therefore, based on the key by Zheng et al. (2025), and disregarding the number of eyes, the adult male of *P. altimontanus* is closest to *P. antennatus* in having the following character states: (i) fore wings developed; (ii) tarsal digitules with pointed apices; (iii) aedeagus blunt apically; and (iv) anterior to procr with more than 5 multilocular pores. The adult male of *P. altimontanus* differs from that of *P. antennatus* by having (contrasting character states in *P. antennatus* are given in parentheses): (i) hamulohalteres with 4 hamuli (hamulohalteres with 1 or 2 hamuli); and (ii) five eyes on each side of the head (with six eyes on each side of head) (data of *P. antennatus* are derived from Reyne (1954)).

## Supplementary Material

XML Treatment for Puto

XML Treatment for Puto altimontanus

## Additional information

### Use of AI

No use of AI was reported.

### Author ORCIDs

Yu-ang Li https://orcid.org/0009-0004-8851-6796

Xinyi Zheng https://orcid.org/0000-0002-7396-7488

Shixiang Zong https://orcid.org/0000-0002-4137-4514

Han Xu https://orcid.org/0000-0001-7226-1742

### Data availability

All of the data that support the findings of this study are available in the main text.
